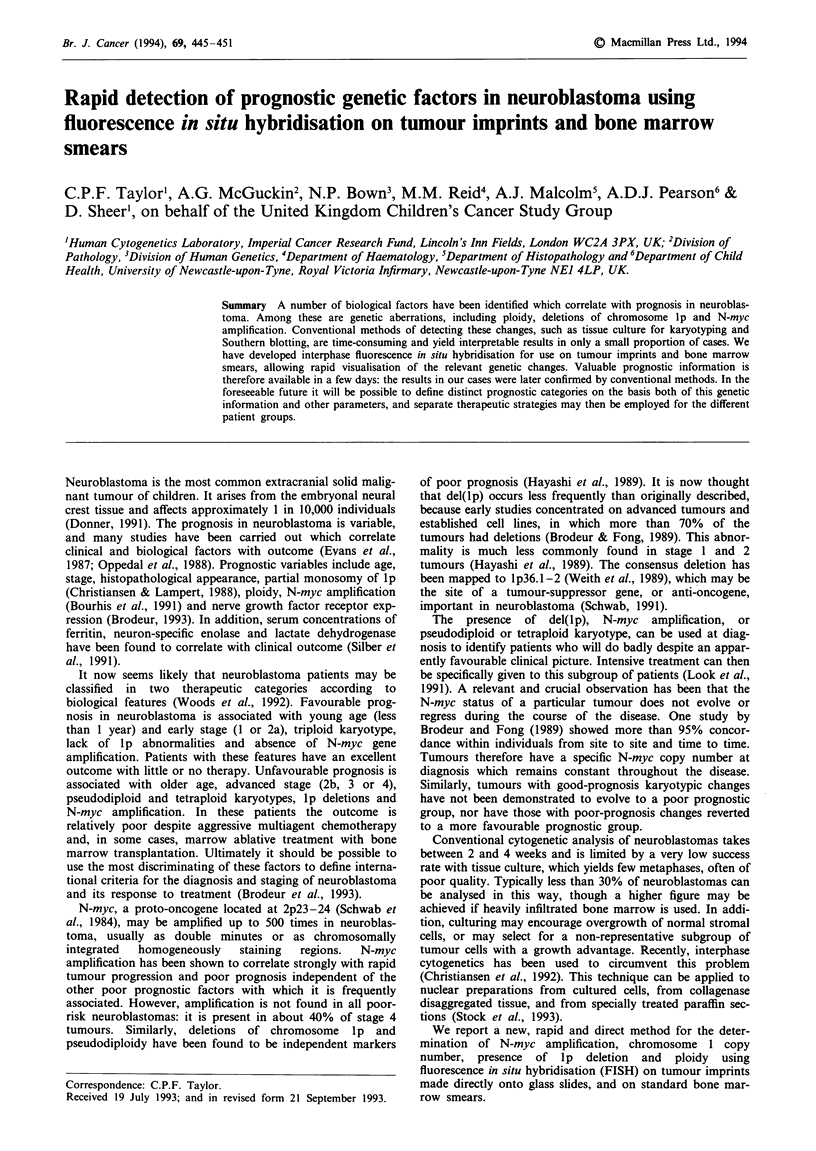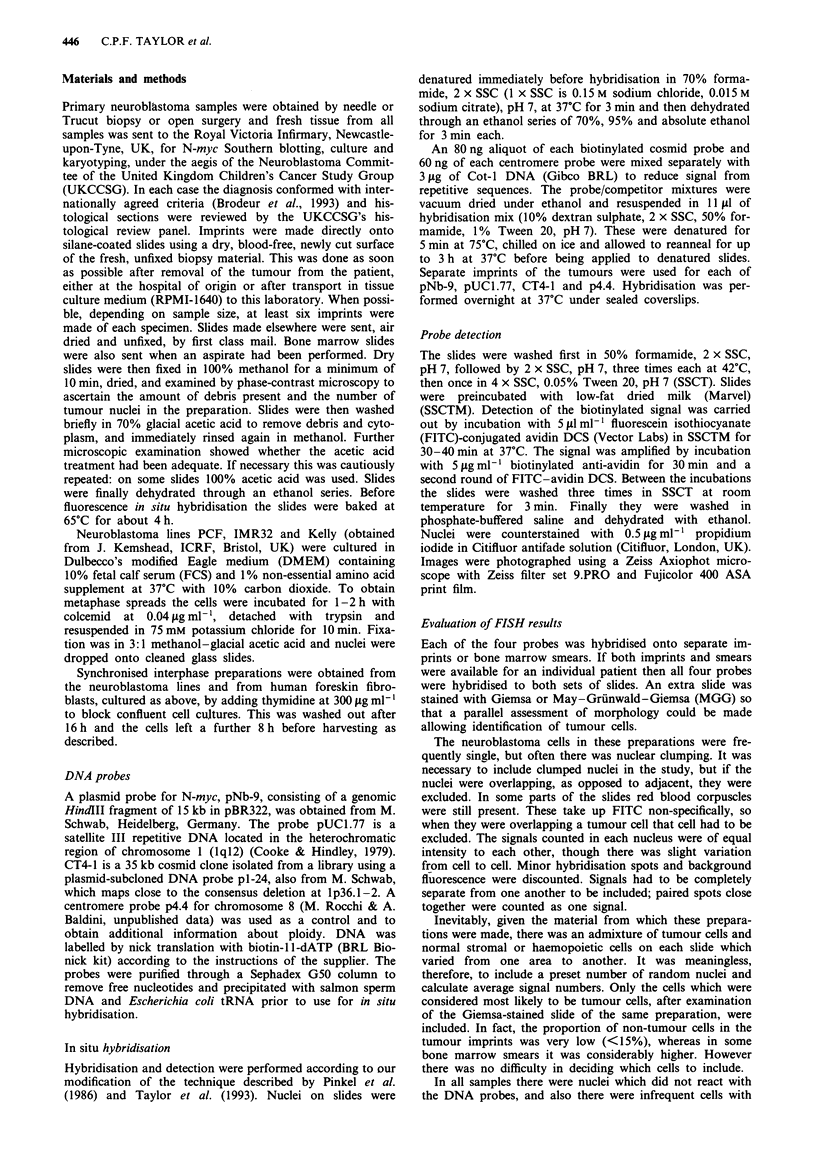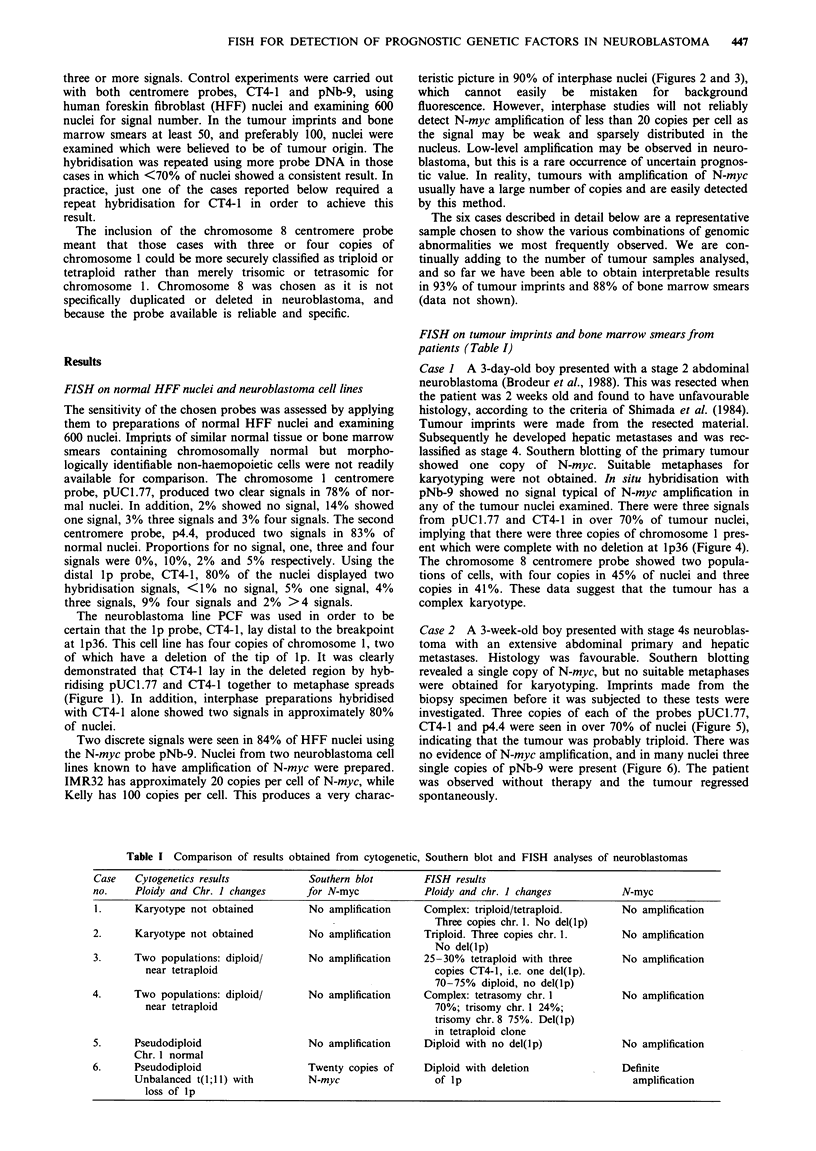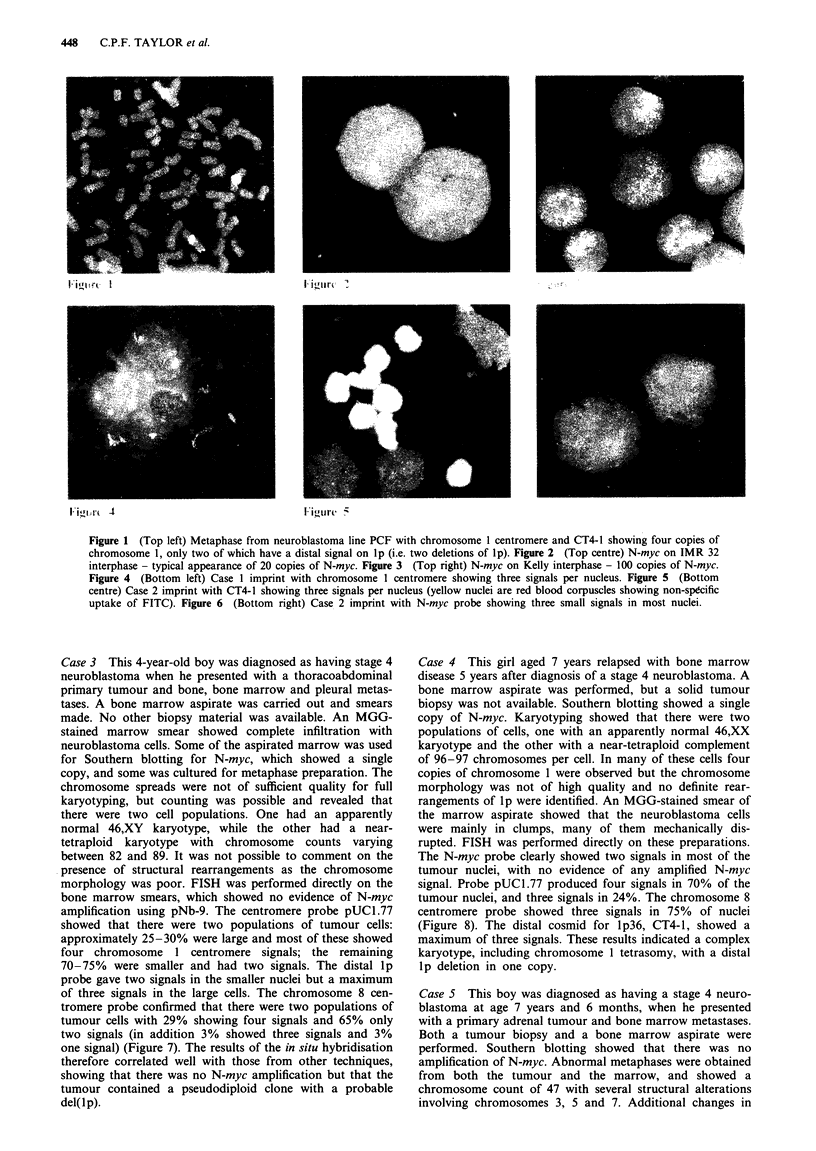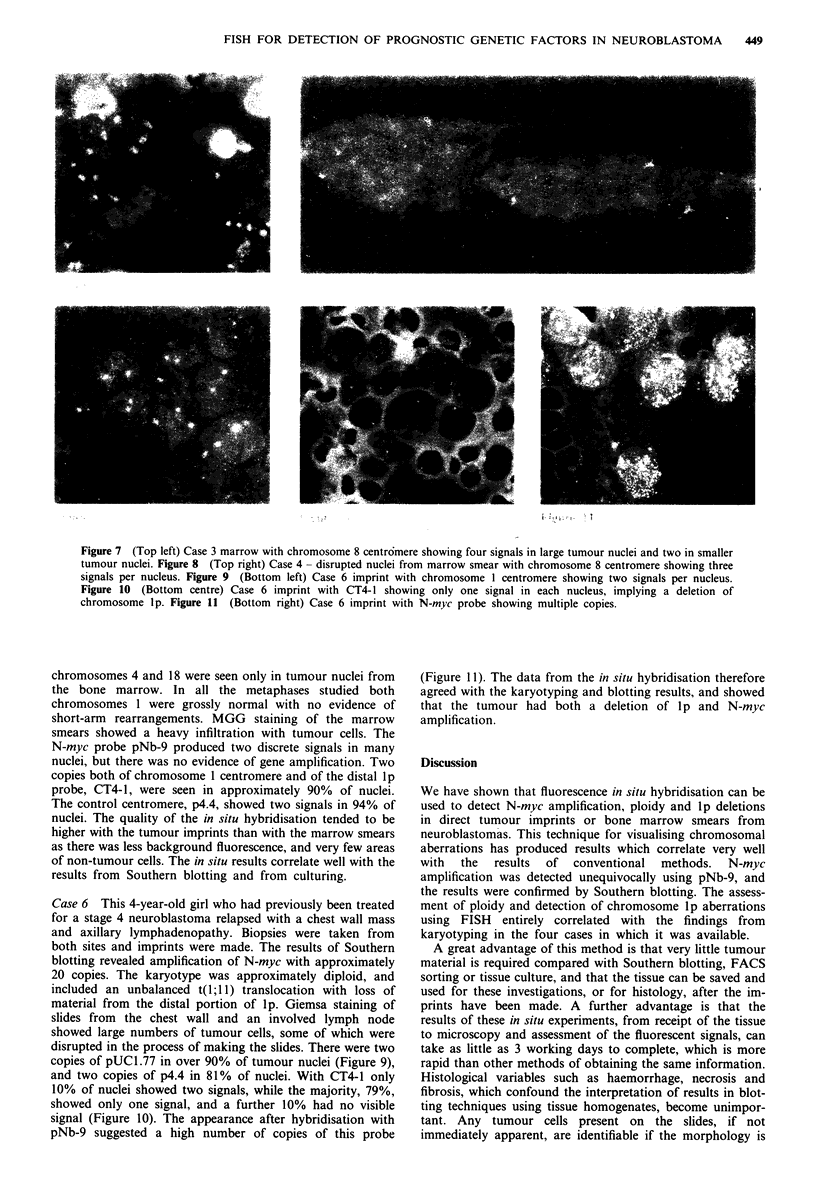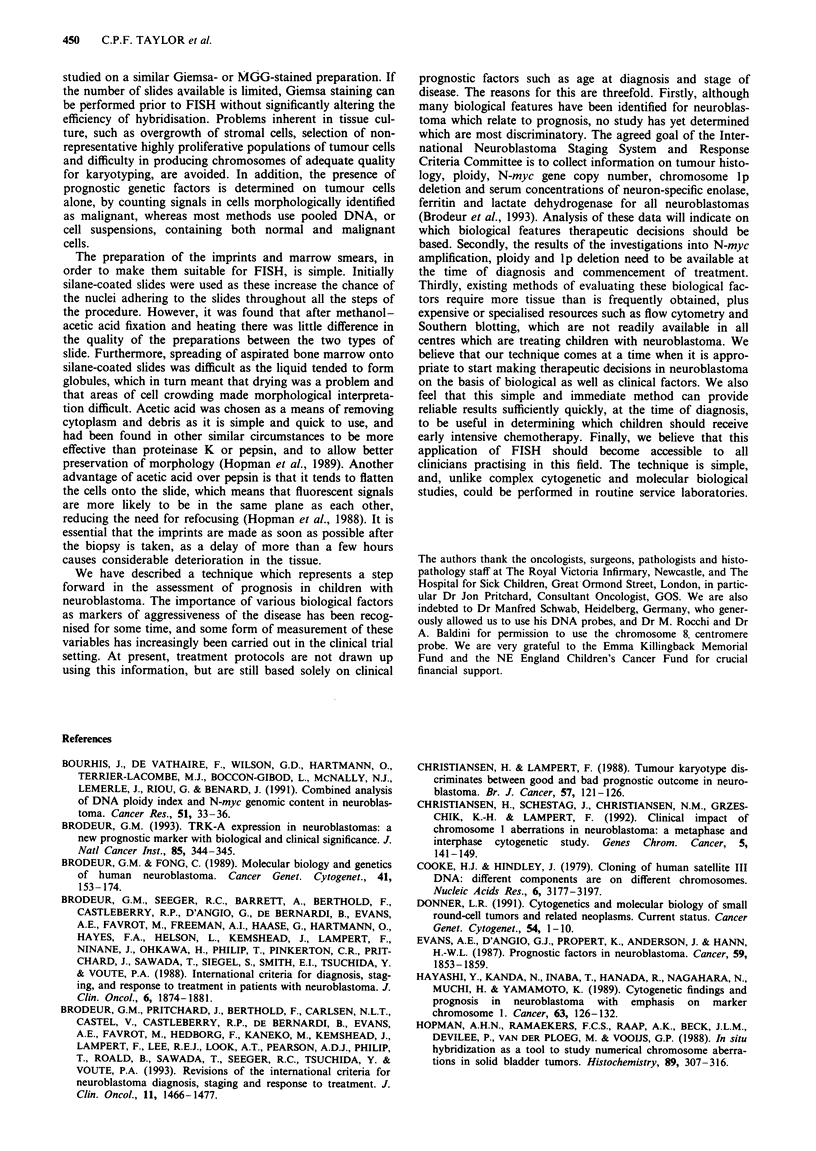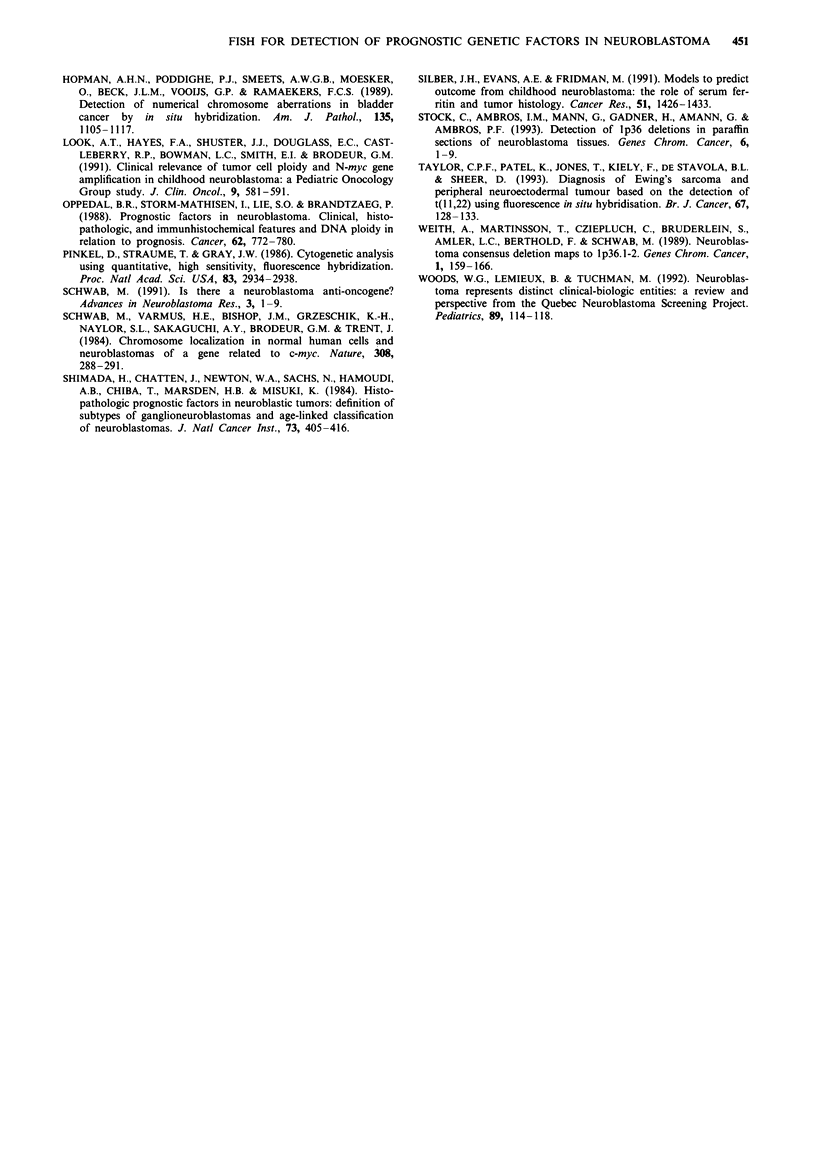# Rapid detection of prognostic genetic factors in neuroblastoma using fluorescence in situ hybridisation on tumour imprints and bone marrow smears. United Kingdom Children's Cancer Study Group.

**DOI:** 10.1038/bjc.1994.81

**Published:** 1994-03

**Authors:** C. P. Taylor, A. G. McGuckin, N. P. Bown, M. M. Reid, A. J. Malcolm, A. D. Pearson, D. Sheer

**Affiliations:** Human Cytogenetics Laboratory, Imperial Cancer Research Fund, London, UK.

## Abstract

**Images:**


Br. J. Cancer (1994), 69, 445-451 © Macmillan Press Ltd., 1994

### Rapid detection of prognostic genetic factors in neuroblastoma using fluorescence *in situ* hybridisation on tumour imprints and bone marrow smears

C.P.F. Taylor[1], A.G. McGuckin[2], N.P. Bown[3], M.M. Reid[4], A.J. Malcolm[5], A.D.J. Pearson[6] & D. Sheer[1], on behalf of the United Kingdom Children's Cancer Study Group

[1]*Human Cytogenetics Laboratory, Imperial Cancer Research Fund, Lincoln's Inn Fields, London WC2A 3PX, UK;* [2]*Division of Pathology,* [3]*Division of Human Genetics,* [4]*Department of Haematology,* [5]*Department of Histopathology and* [6]*Department of Child Health, University of Newcastle-upon-Tyne, Royal Victoria Infirmary, Newcastle-upon-Tyne NE1 4LP, UK.*

**Summary** A number of biological factors have been identified which correlate with prognosis in neuroblastoma. Among these are genetic aberrations, including ploidy, deletions of chromosome 1p and N-*myc* amplification. Conventional methods of detecting these changes, such as tissue culture for karyotyping and Southern blotting, are time-consuming and yield interpretable results in only a small proportion of cases. We have developed interphase fluorescence *in situ* hybridisation for use on tumour imprints and bone marrow smears, allowing rapid visualisation of the relevant genetic changes. Valuable prognostic information is therefore available in a few days: the results in our cases were later confirmed by conventional methods. In the foreseeable future it will be possible to define distinct prognostic categories on the basis both of this genetic information and other parameters, and separate therapeutic strategies may then be employed for the different patient groups.

Neuroblastoma is the most common extracranial solid malignant tumour of children. It arises from the embryonal neural crest tissue and affects approximately 1 in 10,000 individuals (Donner, 1991). The prognosis in neuroblastoma is variable, and many studies have been carried out which correlate clinical and biological factors with outcome (Evans *et al.*, 1987; Oppedal *et al.*, 1988). Prognostic variables include age, stage, histopathological appearance, partial monosomy of 1p (Christiansen & Lampert, 1988), ploidy, N-*myc* amplification (Bourhis *et al.*, 1991) and nerve growth factor receptor expression (Brodeur, 1993). In addition, serum concentrations of ferritin, neuron-specific enolase and lactate dehydrogenase have been found to correlate with clinical outcome (Silber *et al.*, 1991).

It now seems likely that neuroblastoma patients may be classified in two therapeutic categories according to biological features (Woods *et al.*, 1992). Favourable prognosis in neuroblastoma is associated with young age (less than 1 year) and early stage (1 or 2a), triploid karyotype, lack of 1p abnormalities and absence of N-*myc* gene amplification. Patients with these features have an excellent outcome with little or no therapy. Unfavourable prognosis is associated with older age, advanced stage (2b, 3 or 4), pseudodiploid and tetraploid karyotypes, 1p deletions and N-*myc* amplification. In these patients the outcome is relatively poor despite aggressive multiagent chemotherapy and, in some cases, marrow ablative treatment with bone marrow transplantation. Ultimately it should be possible to use the most discriminating of these factors to define international criteria for the diagnosis and staging of neuroblastoma and its response to treatment (Brodeur *et al.*, 1993).

N-*myc*, a proto-oncogene located at 2p23-24 (Schwab *et al.*, 1984), may be amplified up to 500 times in neuroblastoma, usually as double minutes or as chromosomally integrated homogeneously staining regions. N-*myc* amplification has been shown to correlate strongly with rapid tumour progression and poor prognosis independent of the other poor prognostic factors with which it is frequently associated. However, amplification is not found in all poor-risk neuroblastomas: it is present in about 40% of stage 4 tumours. Similarly, deletions of chromosome 1p and pseudodiploidy have been found to be independent markers of poor prognosis (Hayashi *et al.*, 1989). It is now thought that del(1p) occurs less frequently than originally described, because early studies concentrated on advanced tumours and established cell lines, in which more than 70% of the tumours had deletions (Brodeur & Fong, 1989). This abnormality is much less commonly found in stage 1 and 2 tumours (Hayashi *et al.*, 1989). The consensus deletion has been mapped to 1p36.1-2 (Weith *et al.*, 1989), which may be the site of a tumour-suppressor gene, or anti-oncogene, important in neuroblastoma (Schwab, 1991).

The presence of del(1p), N-*myc* amplification, or pseudodiploid or tetraploid karyotype, can be used at diagnosis to identify patients who will do badly despite an apparently favourable clinical picture. Intensive treatment can then be specifically given to this subgroup of patients (Look *et al.*, 1991). A relevant and crucial observation has been that the N-*myc* status of a particular tumour does not evolve or regress during the course of the disease. One study by Brodeur and Fong (1989) showed more than 95% concordance within individuals from site to site and time to time. Tumours therefore have a specific N-*myc* copy number at diagnosis which remains constant throughout the disease. Similarly, tumours with good-prognosis karyotypic changes have not been demonstrated to evolve to a poor prognostic group, nor have those with poor-prognosis changes reverted to a more favourable prognostic group.

Conventional cytogenetic analysis of neuroblastomas takes between 2 and 4 weeks and is limited by a very low success rate with tissue culture, which yields few metaphases, often of poor quality. Typically less than 30% of neuroblastomas can be analysed in this way, though a higher figure may be achieved if heavily infiltrated bone marrow is used. In addition, culturing may encourage overgrowth of normal stromal cells, or may select for a non-representative subgroup of tumour cells with a growth advantage. Recently, interphase cytogenetics has been used to circumvent this problem (Christiansen *et al.*, 1992). This technique can be applied to nuclear preparations from cultured cells, from collagenase disaggregated tissue, and from specially treated paraffin sections (Stock *et al.*, 1993).

We report a new, rapid and direct method for the determination of N-*myc* amplification, chromosome 1 copy number, presence of 1p deletion and ploidy using fluorescence *in situ* hybridisation (FISH) on tumour imprints made directly onto glass slides, and on standard bone marrow smears.

Correspondence: C.P.F. Taylor.
Received 19 July 1993; and in revised form 21 September 1993.

### Materials and methods

Primary neuroblastoma samples were obtained by needle or Trucut biopsy or open surgery and fresh tissue from all samples was sent to the Royal Victoria Infirmary, Newcastle-upon-Tyne, UK, for N-*myc* Southern blotting, culture and karyotyping, under the aegis of the Neuroblastoma Committee of the United Kingdom Children's Cancer Study Group (UKCCSG). In each case the diagnosis conformed with internationally agreed criteria (Brodeur *et al.*, 1993) and histological sections were reviewed by the UKCCSG's histological review panel. Imprints were made directly onto silane-coated slides using a dry, blood-free, newly cut surface of the fresh, unfixed biopsy material. This was done as soon as possible after removal of the tumour from the patient, either at the hospital of origin or after transport in tissue culture medium (RPMI-1640) to this laboratory. When possible, depending on sample size, at least six imprints were made of each specimen. Slides made elsewhere were sent, air dried and unfixed, by first class mail. Bone marrow slides were also sent when an aspirate had been performed. Dry slides were then fixed in 100% methanol for a minimum of 10 min, dried, and examined by phase-contrast microscopy to ascertain the amount of debris present and the number of tumour nuclei in the preparation. Slides were then washed briefly in 70% glacial acetic acid to remove debris and cytoplasm, and immediately rinsed again in methanol. Further microscopic examination showed whether the acetic acid treatment had been adequate. If necessary this was cautiously repeated: on some slides 100% acetic acid was used. Slides were finally dehydrated through an ethanol series. Before fluorescence *in situ* hybridisation the slides were baked at 65°C for about 4 h.

Neuroblastoma lines PCF, IMR32 and Kelly (obtained from J. Kemshead, ICRF, Bristol, UK) were cultured in Dulbecco's modified Eagle medium (DMEM) containing 10% fetal calf serum (FCS) and 1% non-essential amino acid supplement at 37°C with 10% carbon dioxide. To obtain metaphase spreads the cells were incubated for 1–2 h with colcemid at 0.04 $\mu g\ ml^{-1}$, detached with trypsin and resuspended in 75 mM potassium chloride for 10 min. Fixation was in 3:1 methanol–glacial acetic acid and nuclei were dropped onto cleaned glass slides.

Synchronised interphase preparations were obtained from the neuroblastoma lines and from human foreskin fibroblasts, cultured as above, by adding thymidine at 300 $\mu g\ ml^{-1}$ to block confluent cell cultures. This was washed out after 16 h and the cells left a further 8 h before harvesting as described.

### *DNA probes*

A plasmid probe for N-*myc*, pNb-9, consisting of a genomic *Hind*III fragment of 15 kb in pBR322, was obtained from M. Schwab, Heidelberg, Germany. The probe pUC1.77 is a satellite III repetitive DNA located in the heterochromatic region of chromosome 1 (1q12) (Cooke & Hindley, 1979). CT4-1 is a 35 kb cosmid clone isolated from a library using a plasmid-subcloned DNA probe p1-24, also from M. Schwab, which maps close to the consensus deletion at 1p36.1–2. A centromere probe p4.4 for chromosome 8 (M. Rocchi & A. Baldini, unpublished data) was used as a control and to obtain additional information about ploidy. DNA was labelled by nick translation with biotin-11-dATP (BRL Bionick kit) according to the instructions of the supplier. The probes were purified through a Sephadex G50 column to remove free nucleotides and precipitated with salmon sperm DNA and *Escherichia coli* tRNA prior to use for *in situ* hybridisation.

### In situ *hybridisation*

Hybridisation and detection were performed according to our modification of the technique described by Pinkel *et al.* (1986) and Taylor *et al.* (1993). Nuclei on slides were denatured immediately before hybridisation in 70% formamide, 2 × SSC (1 × SSC is 0.15 M sodium chloride, 0.015 M sodium citrate), pH 7, at 37°C for 3 min and then dehydrated through an ethanol series of 70%, 95% and absolute ethanol for 3 min each.

An 80 ng aliquot of each biotinylated cosmid probe and 60 ng of each centromere probe were mixed separately with 3 $\mu g$ of Cot-1 DNA (Gibco BRL) to reduce signal from repetitive sequences. The probe/competitor mixtures were vacuum dried under ethanol and resuspended in 11 $\mu l$ of hybridisation mix (10% dextran sulphate, 2 × SSC, 50% formamide, 1% Tween 20, pH 7). These were denatured for 5 min at 75°C, chilled on ice and allowed to reanneal for up to 3 h at 37°C before being applied to denatured slides. Separate imprints of the tumours were used for each of pNb-9, pUC1.77, CT4-1 and p4.4. Hybridisation was performed overnight at 37°C under sealed coverslips.

### *Probe detection*

The slides were washed first in 50% formamide, 2 × SSC, pH 7, followed by 2 × SSC, pH 7, three times each at 42°C, then once in 4 × SSC, 0.05% Tween 20, pH 7 (SSCT). Slides were preincubated with low-fat dried milk (Marvel) (SSCTM). Detection of the biotinylated signal was carried out by incubation with 5 $\mu l\ ml^{-1}$ fluorescein isothiocyanate (FITC)-conjugated avidin DCS (Vector Labs) in SSCTM for 30–40 min at 37°C. The signal was amplified by incubation with 5 $\mu g\ ml^{-1}$ biotinylated anti-avidin for 30 min and a second round of FITC–avidin DCS. Between the incubations the slides were washed three times in SSCT at room temperature for 3 min. Finally they were washed in phosphate-buffered saline and dehydrated with ethanol. Nuclei were counterstained with 0.5 $\mu g\ ml^{-1}$ propidium iodide in Citifluor antifade solution (Citifluor, London, UK). Images were photographed using a Zeiss Axiophot microscope with Zeiss filter set 9.PRO and Fujicolor 400 ASA print film.

### *Evaluation of FISH results*

Each of the four probes was hybridised onto separate imprints or bone marrow smears. If both imprints and smears were available for an individual patient then all four probes were hybridised to both sets of slides. An extra slide was stained with Giemsa or May–Grünwald–Giemsa (MGG) so that a parallel assessment of morphology could be made allowing identification of tumour cells.

The neuroblastoma cells in these preparations were frequently single, but often there was nuclear clumping. It was necessary to include clumped nuclei in the study, but if the nuclei were overlapping, as opposed to adjacent, they were excluded. In some parts of the slides red blood corpuscles were still present. These take up FITC non-specifically, so when they were overlapping a tumour cell that cell had to be excluded. The signals counted in each nucleus were of equal intensity to each other, though there was slight variation from cell to cell. Minor hybridisation spots and background fluorescence were discounted. Signals had to be completely separate from one another to be included; paired spots close together were counted as one signal.

Inevitably, given the material from which these preparations were made, there was an admixture of tumour cells and normal stromal or haemopoietic cells on each slide which varied from one area to another. It was meaningless, therefore, to include a preset number of random nuclei and calculate average signal numbers. Only the cells which were considered most likely to be tumour cells, after examination of the Giemsa-stained slide of the same preparation, were included. In fact, the proportion of non-tumour cells in the tumour imprints was very low (<15%), whereas in some bone marrow smears it was considerably higher. However there was no difficulty in deciding which cells to include.

In all samples there were nuclei which did not react with the DNA probes, and also there were infrequent cells with three or more signals. Control experiments were carried out with both centromere probes, CT4-1 and pNb-9, using human foreskin fibroblast (HFF) nuclei and examining 600 nuclei for signal number. In the tumour imprints and bone marrow smears at least 50, and preferably 100, nuclei were examined which were believed to be of tumour origin. The hybridisation was repeated using more probe DNA in those cases in which <70% of nuclei showed a consistent result. In practice, just one of the cases reported below required a repeat hybridisation for CT4-1 in order to achieve this result.

The inclusion of the chromosome 8 centromere probe meant that those cases with three or four copies of chromosome 1 could be more securely classified as triploid or tetraploid rather than merely trisomic or tetrasomic for chromosome 1. Chromosome 8 was chosen as it is not specifically duplicated or deleted in neuroblastoma, and because the probe available is reliable and specific.

### Results

### *FISH on normal HFF nuclei and neuroblastoma cell lines*

The sensitivity of the chosen probes was assessed by applying them to preparations of normal HFF nuclei and examining 600 nuclei. Imprints of similar normal tissue or bone marrow smears containing chromosomally normal but morphologically identifiable non-haemopoietic cells were not readily available for comparison. The chromosome 1 centromere probe, pUC1.77, produced two clear signals in 78% of normal nuclei. In addition, 2% showed no signal, 14% showed one signal, 3% three signals and 3% four signals. The second centromere probe, p4.4, produced two signals in 83% of normal nuclei. Proportions for no signal, one, three and four signals were 0%, 10%, 2% and 5% respectively. Using the distal 1p probe, CT4-1, 80% of the nuclei displayed two hybridisation signals, <1% no signal, 5% one signal, 4% three signals, 9% four signals and 2% >4 signals.

The neuroblastoma line PCF was used in order to be certain that the 1p probe, CT4-1, lay distal to the breakpoint at 1p36. This cell line has four copies of chromosome 1, two of which have a deletion of the tip of 1p. It was clearly demonstrated that CT4-1 lay in the deleted region by hybridising pUC1.77 and CT4-1 together to metaphase spreads (Figure 1). In addition, interphase preparations hybridised with CT4-1 alone showed two signals in approximately 80% of nuclei.

Two discrete signals were seen in 84% of HFF nuclei using the N-*myc* probe pNb-9. Nuclei from two neuroblastoma cell lines known to have amplification of N-*myc* were prepared. IMR32 has approximately 20 copies per cell of N-*myc*, while Kelly has 100 copies per cell. This produces a very characteristic picture in 90% of interphase nuclei (Figures 2 and 3), which cannot easily be mistaken for background fluorescence. However, interphase studies will not reliably detect N-*myc* amplification of less than 20 copies per cell as the signal may be weak and sparsely distributed in the nucleus. Low-level amplification may be observed in neuroblastoma, but this is a rare occurrence of uncertain prognostic value. In reality, tumours with amplification of N-*myc* usually have a large number of copies and are easily detected by this method.

The six cases described in detail below are a representative sample chosen to show the various combinations of genomic abnormalities we most frequently observed. We are continually adding to the number of tumour samples analysed, and so far we have been able to obtain interpretable results in 93% of tumour imprints and 88% of bone marrow smears (data not shown).

### *FISH on tumour imprints and bone marrow smears from patients (Table I)*

*Case 1* A 3-day-old boy presented with a stage 2 abdominal neuroblastoma (Brodeur *et al.*, 1988). This was resected when the patient was 2 weeks old and found to have unfavourable histology, according to the criteria of Shimada *et al.* (1984). Tumour imprints were made from the resected material. Subsequently he developed hepatic metastases and was reclassified as stage 4. Southern blotting of the primary tumour showed one copy of N-*myc*. Suitable metaphases for karyotyping were not obtained. *In situ* hybridisation with pNb-9 showed no signal typical of N-*myc* amplification in any of the tumour nuclei examined. There were three signals from pUC1.77 and CT4-1 in over 70% of tumour nuclei, implying that there were three copies of chromosome 1 present which were complete with no deletion at 1p36 (Figure 4). The chromosome 8 centromere probe showed two populations of cells, with four copies in 45% of nuclei and three copies in 41%. These data suggest that the tumour has a complex karyotype.

*Case 2* A 3-week-old boy presented with stage 4s neuroblastoma with an extensive abdominal primary and hepatic metastases. Histology was favourable. Southern blotting revealed a single copy of N-*myc*, but no suitable metaphases were obtained for karyotyping. Imprints made from the biopsy specimen before it was subjected to these tests were investigated. Three copies of each of the probes pUC1.77, CT4-1 and p4.4 were seen in over 70% of nuclei (Figure 5), indicating that the tumour was probably triploid. There was no evidence of N-*myc* amplification, and in many nuclei three single copies of pNb-9 were present (Figure 6). The patient was observed without therapy and the tumour regressed spontaneously.

**Table I** Comparison of results obtained from cytogenetic, Southern blot and FISH analyses of neuroblastomas

| *Case no.* | *Cytogenetics results Ploidy and Chr. 1 changes* | *Southern blot for* N-myc | *FISH results Ploidy and chr. 1 changes* | *N*-myc |
|---|---|---|---|---|
| 1. | Karyotype not obtained | No amplification | Complex: triploid/tetraploid. Three copies chr. 1. No del(1p) | No amplification |
| 2. | Karyotype not obtained | No amplification | Triploid. Three copies chr. 1. No del(1p) | No amplification |
| 3. | Two populations: diploid/ near tetraploid | No amplification | 25–30% tetraploid with three copies CT4-1, i.e. one del(1p). 70–75% diploid, no del(1p) | No amplification |
| 4. | Two populations: diploid/ near tetraploid | No amplification | Complex: tetrasomy chr. 1 70%; trisomy chr. 1 24%; trisomy chr. 8 75%. Del(1p) in tetraploid clone | No amplification |
| 5. | Pseudodiploid Chr. 1 normal | No amplification | Diploid with no del(1p) | No amplification |
| 6. | Pseudodiploid Unbalanced t(1;11) with loss of 1p | Twenty copies of N-*myc* | Diploid with deletion of 1p | Definite amplification |

Figure 1

Figure 2

Figure 3

Figure 4

Figure 5

Figure 6

**Figure 1** (Top left) Metaphase from neuroblastoma line PCF with chromosome 1 centromere and CT4-1 showing four copies of chromosome 1, only two of which have a distal signal on 1p (i.e. two deletions of 1p). **Figure 2** (Top centre) N-*myc* on IMR 32 interphase – typical appearance of 20 copies of N-*myc*. **Figure 3** (Top right) N-*myc* on Kelly interphase – 100 copies of N-*myc*. **Figure 4** (Bottom left) Case 1 imprint with chromosome 1 centromere showing three signals per nucleus. **Figure 5** (Bottom centre) Case 2 imprint with CT4-1 showing three signals per nucleus (yellow nuclei are red blood corpuscles showing non-specific uptake of FITC). **Figure 6** (Bottom right) Case 2 imprint with N-*myc* probe showing three small signals in most nuclei.

*Case 3* This 4-year-old boy was diagnosed as having stage 4 neuroblastoma when he presented with a thoracoabdominal primary tumour and bone, bone marrow and pleural metastases. A bone marrow aspirate was carried out and smears made. No other biopsy material was available. An MGG-stained marrow smear showed complete infiltration with neuroblastoma cells. Some of the aspirated marrow was used for Southern blotting for N-*myc*, which showed a single copy, and some was cultured for metaphase preparation. The chromosome spreads were not of sufficient quality for full karyotyping, but counting was possible and revealed that there were two cell populations. One had an apparently normal 46,XY karyotype, while the other had a near-tetraploid karyotype with chromosome counts varying between 82 and 89. It was not possible to comment on the presence of structural rearrangements as the chromosome morphology was poor. FISH was performed directly on the bone marrow smears, which showed no evidence of N-*myc* amplification using pNb-9. The centromere probe pUC1.77 showed that there were two populations of tumour cells: approximately 25–30% were large and most of these showed four chromosome 1 centromere signals; the remaining 70–75% were smaller and had two signals. The distal 1p probe gave two signals in the smaller nuclei but a maximum of three signals in the large cells. The chromosome 8 centromere probe confirmed that there were two populations of tumour cells with 29% showing four signals and 65% only two signals (in addition 3% showed three signals and 3% one signal) (Figure 7). The results of the *in situ* hybridisation therefore correlated well with those from other techniques, showing that there was no N-*myc* amplification but that the tumour contained a pseudodiploid clone with a probable del(1p).

*Case 4* This girl aged 7 years relapsed with bone marrow disease 5 years after diagnosis of a stage 4 neuroblastoma. A bone marrow aspirate was performed, but a solid tumour biopsy was not available. Southern blotting showed a single copy of N-*myc*. Karyotyping showed that there were two populations of cells, one with an apparently normal 46,XX karyotype and the other with a near-tetraploid complement of 96–97 chromosomes per cell. In many of these cells four copies of chromosome 1 were observed but the chromosome morphology was not of high quality and no definite rearrangements of 1p were identified. An MGG-stained smear of the marrow aspirate showed that the neuroblastoma cells were mainly in clumps, many of them mechanically disrupted. FISH was performed directly on these preparations. The N-*myc* probe clearly showed two signals in most of the tumour nuclei, with no evidence of any amplified N-*myc* signal. Probe pUC1.77 produced four signals in 70% of the tumour nuclei, and three signals in 24%. The chromosome 8 centromere probe showed three signals in 75% of nuclei (Figure 8). The distal cosmid for 1p36, CT4-1, showed a maximum of three signals. These results indicated a complex karyotype, including chromosome 1 tetrasomy, with a distal 1p deletion in one copy.

*Case 5* This boy was diagnosed as having a stage 4 neuroblastoma at age 7 years and 6 months, when he presented with a primary adrenal tumour and bone marrow metastases. Both a tumour biopsy and a bone marrow aspirate were performed. Southern blotting showed that there was no amplification of N-*myc*. Abnormal metaphases were obtained from both the tumour and the marrow, and showed a chromosome count of 47 with several structural alterations involving chromosomes 3, 5 and 7. Additional changes in

Figure 7

Figure 8

Figure 9

Figure 10

Figure 11

**Figure 7** (Top left) Case 3 marrow with chromosome 8 centromere showing four signals in large tumour nuclei and two in smaller tumour nuclei. **Figure 8** (Top right) Case 4 – disrupted nuclei from marrow smear with chromosome 8 centromere showing three signals per nucleus. **Figure 9** (Bottom left) Case 6 imprint with chromosome 1 centromere showing two signals per nucleus. **Figure 10** (Bottom centre) Case 6 imprint with CT4-1 showing only one signal in each nucleus, implying a deletion of chromosome 1p. **Figure 11** (Bottom right) Case 6 imprint with N-*myc* probe showing multiple copies.

chromosomes 4 and 18 were seen only in tumour nuclei from the bone marrow. In all the metaphases studied both chromosomes 1 were grossly normal with no evidence of short-arm rearrangements. MGG staining of the marrow smears showed a heavy infiltration with tumour cells. The N-*myc* probe pNb-9 produced two discrete signals in many nuclei, but there was no evidence of gene amplification. Two copies both of chromosome 1 centromere and of the distal 1p probe, CT4-1, were seen in approximately 90% of nuclei. The control centromere, p4.4, showed two signals in 94% of nuclei. The quality of the *in situ* hybridisation tended to be higher with the tumour imprints than with the marrow smears as there was less background fluorescence, and very few areas of non-tumour cells. The *in situ* results correlate well with the results from Southern blotting and from culturing.

*Case 6* This 4-year-old girl who had previously been treated for a stage 4 neuroblastoma relapsed with a chest wall mass and axillary lymphadenopathy. Biopsies were taken from both sites and imprints were made. The results of Southern blotting revealed amplification of N-*myc* with approximately 20 copies. The karyotype was approximately diploid, and included an unbalanced t(1;11) translocation with loss of material from the distal portion of 1p. Giemsa staining of slides from the chest wall and an involved lymph node showed large numbers of tumour cells, some of which were disrupted in the process of making the slides. There were two copies of pUC1.77 in over 90% of tumour nuclei (Figure 9), and two copies of p4.4 in 81% of nuclei. With CT4-1 only 10% of nuclei showed two signals, while the majority, 79%, showed only one signal, and a further 10% had no visible signal (Figure 10). The appearance after hybridisation with pNb-9 suggested a high number of copies of this probe (Figure 11). The data from the *in situ* hybridisation therefore agreed with the karyotyping and blotting results, and showed that the tumour had both a deletion of 1p and N-*myc* amplification.

### Discussion

We have shown that fluorescence *in situ* hybridisation can be used to detect N-*myc* amplification, ploidy and 1p deletions in direct tumour imprints or bone marrow smears from neuroblastomas. This technique for visualising chromosomal aberrations has produced results which correlate very well with the results of conventional methods. N-*myc* amplification was detected unequivocally using pNb-9, and the results were confirmed by Southern blotting. The assessment of ploidy and detection of chromosome 1p aberrations using FISH entirely correlated with the findings from karyotyping in the four cases in which it was available.

A great advantage of this method is that very little tumour material is required compared with Southern blotting, FACS sorting or tissue culture, and that the tissue can be saved and used for these investigations, or for histology, after the imprints have been made. A further advantage is that the results of these *in situ* experiments, from receipt of the tissue to microscopy and assessment of the fluorescent signals, can take as little as 3 working days to complete, which is more rapid than other methods of obtaining the same information. Histological variables such as haemorrhage, necrosis and fibrosis, which confound the interpretation of results in blotting techniques using tissue homogenates, become unimportant. Any tumour cells present on the slides, if not immediately apparent, are identifiable if the morphology is studied on a similar Giemsa- or MGG-stained preparation. If the number of slides available is limited, Giemsa staining can be performed prior to FISH without significantly altering the efficiency of hybridisation. Problems inherent in tissue culture, such as overgrowth of stromal cells, selection of non-representative highly proliferative populations of tumour cells and difficulty in producing chromosomes of adequate quality for karyotyping, are avoided. In addition, the presence of prognostic genetic factors is determined on tumour cells alone, by counting signals in cells morphologically identified as malignant, whereas most methods use pooled DNA, or cell suspensions, containing both normal and malignant cells.

The preparation of the imprints and marrow smears, in order to make them suitable for FISH, is simple. Initially silane-coated slides were used as these increase the chance of the nuclei adhering to the slides throughout all the steps of the procedure. However, it was found that after methanol–acetic acid fixation and heating there was little difference in the quality of the preparations between the two types of slide. Furthermore, spreading of aspirated bone marrow onto silane-coated slides was difficult as the liquid tended to form globules, which in turn meant that drying was a problem and that areas of cell crowding made morphological interpretation difficult. Acetic acid was chosen as a means of removing cytoplasm and debris as it is simple and quick to use, and had been found in other similar circumstances to be more effective than proteinase K or pepsin, and to allow better preservation of morphology (Hopman *et al.*, 1989). Another advantage of acetic acid over pepsin is that it tends to flatten the cells onto the slide, which means that fluorescent signals are more likely to be in the same plane as each other, reducing the need for refocusing (Hopman *et al.*, 1988). It is essential that the imprints are made as soon as possible after the biopsy is taken, as a delay of more than a few hours causes considerable deterioration in the tissue.

We have described a technique which represents a step forward in the assessment of prognosis in children with neuroblastoma. The importance of various biological factors as markers of aggressiveness of the disease has been recognised for some time, and some form of measurement of these variables has increasingly been carried out in the clinical trial setting. At present, treatment protocols are not drawn up using this information, but are still based solely on clinical prognostic factors such as age at diagnosis and stage of disease. The reasons for this are threefold. Firstly, although many biological features have been identified for neuroblastoma which relate to prognosis, no study has yet determined which are most discriminatory. The agreed goal of the International Neuroblastoma Staging System and Response Criteria Committee is to collect information on tumour histology, ploidy, N-*myc* gene copy number, chromosome 1p deletion and serum concentrations of neuron-specific enolase, ferritin and lactate dehydrogenase for all neuroblastomas (Brodeur *et al.*, 1993). Analysis of these data will indicate on which biological features therapeutic decisions should be based. Secondly, the results of the investigations into N-*myc* amplification, ploidy and 1p deletion need to be available at the time of diagnosis and commencement of treatment. Thirdly, existing methods of evaluating these biological factors require more tissue than is frequently obtained, plus expensive or specialised resources such as flow cytometry and Southern blotting, which are not readily available in all centres which are treating children with neuroblastoma. We believe that our technique comes at a time when it is appropriate to start making therapeutic decisions in neuroblastoma on the basis of biological as well as clinical factors. We also feel that this simple and immediate method can provide reliable results sufficiently quickly, at the time of diagnosis, to be useful in determining which children should receive early intensive chemotherapy. Finally, we believe that this application of FISH should become accessible to all clinicians practising in this field. The technique is simple, and, unlike complex cytogenetic and molecular biological studies, could be performed in routine service laboratories.

The authors thank the oncologists, surgeons, pathologists and histopathology staff at The Royal Victoria Infirmary, Newcastle, and The Hospital for Sick Children, Great Ormond Street, London, in particular Dr Jon Pritchard, Consultant Oncologist, GOS. We are also indebted to Dr Manfred Schwab, Heidelberg, Germany, who generously allowed us to use his DNA probes, and Dr M. Rocchi and Dr A. Baldini for permission to use the chromosome 8 centromere probe. We are very grateful to the Emma Killingback Memorial Fund and the NE England Children's Cancer Fund for crucial financial support.

### References

BOURHIS, J., DE VATHAIRE, F., WILSON, G.D., HARTMANN, O., TERRIER-LACOMBE, M.J., BOCCON-GIBOD, L., MCNALLY, N.J., LEMERLE, J., RIOU, G. & BENARD, J. (1991). Combined analysis of DNA ploidy index and N-*myc* genomic content in neuroblastoma. *Cancer Res.*, **51**, 33–36.

BRODEUR, G.M. (1993). TRK-A expression in neuroblastomas: a new prognostic marker with biological and clinical significance. *J. Natl Cancer Inst.*, **85**, 344–345.

BRODEUR, G.M. & FONG, C. (1989). Molecular biology and genetics of human neuroblastoma. *Cancer Genet. Cytogenet.*, **41**, 153–174.

BRODEUR, G.M., SEEGER, R.C., BARRETT, A., BERTHOLD, F., CASTLEBERRY, R.P., D'ANGIO, G., DE BERNARDI, B., EVANS, A.E., FAVROT, M., FREEMAN, A.I., HAASE, G., HARTMANN, O., HAYES, F.A., HELSON, L., KEMSHEAD, J., LAMPERT, F., NINANE, J., OHKAWA, H., PHILIP, T., PINKERTON, C.R., PRITCHARD, J., SAWADA, T., SIEGEL, S., SMITH, E.I., TSUCHIDA, Y. & VOUTE, P.A. (1988). International criteria for diagnosis, staging, and response to treatment in patients with neuroblastoma. *J. Clin. Oncol.*, **6**, 1874–1881.

BRODEUR, G.M., PRITCHARD, J., BERTHOLD, F., CARLSEN, N.L.T., CASTEL, V., CASTLEBERRY, R.P., DE BERNARDI, B., EVANS, A.E., FAVROT, M., HEDBORG, F., KANEKO, M., KEMSHEAD, J., LAMPERT, F., LEE, R.E.J., LOOK, A.T., PEARSON, A.D.J., PHILIP, T., ROALD, B., SAWADA, T., SEEGER, R.C., TSUCHIDA, Y. & VOUTE, P.A. (1993). Revisions of the international criteria for neuroblastoma diagnosis, staging and response to treatment. *J. Clin. Oncol.*, **11**, 1466–1477.

CHRISTIANSEN, H. & LAMPERT, F. (1988). Tumour karyotype discriminates between good and bad prognostic outcome in neuroblastoma. *Br. J. Cancer*, **57**, 121–126.

CHRISTIANSEN, H., SCHESTAG, J., CHRISTIANSEN, N.M., GRZESCHIK, K.-H. & LAMPERT, F. (1992). Clinical impact of chromosome 1 aberrations in neuroblastoma: a metaphase and interphase cytogenetic study. *Genes Chrom. Cancer*, **5**, 141–149.

COOKE, H.J. & HINDLEY, J. (1979). Cloning of human satellite III DNA: different components are on different chromosomes. *Nucleic Acids Res.*, **6**, 3177–3197.

DONNER, L.R. (1991). Cytogenetics and molecular biology of small round-cell tumors and related neoplasms. Current status. *Cancer Genet. Cytogenet.*, **54**, 1–10.

EVANS, A.E., D'ANGIO, G.J., PROPERT, K., ANDERSON, J. & HANN, H.-W.L. (1987). Prognostic factors in neuroblastoma. *Cancer*, **59**, 1853–1859.

HAYASHI, Y., KANDA, N., INABA, T., HANADA, R., NAGAHARA, N., MUCHI, H. & YAMAMOTO, K. (1989). Cytogenetic findings and prognosis in neuroblastoma with emphasis on marker chromosome 1. *Cancer*, **63**, 126–132.

HOPMAN, A.H.N., RAMAEKERS, F.C.S., RAAP, A.K., BECK, J.L.M., DEVILEE, P., VAN DER PLOEG, M. & VOOIJS, G.P. (1988). *In situ* hybridization as a tool to study numerical chromosome aberrations in solid bladder tumors. *Histochemistry*, **89**, 307–316.

HOPMAN, A.H.N., PODDIGHE, P.J., SMEETS, A.W.G.B., MOESKER, O., BECK, J.L.M., VOOIJS, G.P. & RAMAEKERS, F.C.S. (1989). Detection of numerical chromosome aberrations in bladder cancer by *in situ* hybridization. *Am. J. Pathol.*, **135**, 1105–1117.

LOOK, A.T., HAYES, F.A., SHUSTER, J.J., DOUGLASS, E.C., CASTLEBERRY, R.P., BOWMAN, L.C., SMITH, E.I. & BRODEUR, G.M. (1991). Clinical relevance of tumor cell ploidy and N-*myc* gene amplification in childhood neuroblastoma: a Pediatric Onocology Group study. *J. Clin. Oncol.*, **9**, 581–591.

OPPEDAL, B.R., STORM-MATHISEN, I., LIE, S.O. & BRANDTZAEG, P. (1988). Prognostic factors in neuroblastoma. Clinical, histopathologic, and immunhistochemical features and DNA ploidy in relation to prognosis. *Cancer*, **62**, 772–780.

PINKEL, D., STRAUME, T. & GRAY, J.W. (1986). Cytogenetic analysis using quantitative, high sensitivity, fluorescence hybridization. *Proc. Natl Acad. Sci. USA*, **83**, 2934–2938.

SCHWAB, M. (1991). Is there a neuroblastoma anti-oncogene? *Advances in Neuroblastoma Res.*, **3**, 1–9.

SCHWAB, M., VARMUS, H.E., BISHOP, J.M., GRZESCHIK, K.-H., NAYLOR, S.L., SAKAGUCHI, A.Y., BRODEUR, G.M. & TRENT, J. (1984). Chromosome localization in normal human cells and neuroblastomas of a gene related to c-*myc*. *Nature*, **308**, 288–291.

SHIMADA, H., CHATTEN, J., NEWTON, W.A., SACHS, N., HAMOUDI, A.B., CHIBA, T., MARSDEN, H.B. & MISUKI, K. (1984). Histopathologic prognostic factors in neuroblastic tumors: definition of subtypes of ganglioneuroblastomas and age-linked classification of neuroblastomas. *J. Natl Cancer Inst.*, **73**, 405–416.

SILBER, J.H., EVANS, A.E. & FRIDMAN, M. (1991). Models to predict outcome from childhood neuroblastoma: the role of serum ferritin and tumor histology. *Cancer Res.*, **51**, 1426–1433.

STOCK, C., AMBROS, I.M., MANN, G., GADNER, H., AMANN, G. & AMBROS, P.F. (1993). Detection of 1p36 deletions in paraffin sections of neuroblastoma tissues. *Genes Chrom. Cancer*, **6**, 1–9.

TAYLOR, C.P.F., PATEL, K., JONES, T., KIELY, F., DE STAVOLA, B.L. & SHEER, D. (1993). Diagnosis of Ewing's sarcoma and peripheral neuroectodermal tumour based on the detection of t(11,22) using fluorescence *in situ* hybridisation. *Br. J. Cancer*, **67**, 128–133.

WEITH, A., MARTINSSON, T., CZIEPLUCH, C., BRUDERLEIN, S., AMLER, L.C., BERTHOLD, F. & SCHWAB, M. (1989). Neuroblastoma consensus deletion maps to 1p36.1-2. *Genes Chrom. Cancer*, **1**, 159–166.

WOODS, W.G., LEMIEUX, B. & TUCHMAN, M. (1992). Neuroblastoma represents distinct clinical-biologic entities: a review and perspective from the Quebec Neuroblastoma Screening Project. *Pediatrics*, **89**, 114–118.